# Predictors of Mortality in Adults With Sickle Cell Disease Admitted to the Intensive Care Unit in King Saud Medical City, Saudi Arabia

**DOI:** 10.7759/cureus.38817

**Published:** 2023-05-10

**Authors:** Mustafa Alhaj Zeen, Nourhan E Mohamed, Ahmed F Mady, Mohammed M Alamri, Saitah Alshammari, Abdulilh K Alshebaily, Huda Hijazi, Asmaa Hegazy

**Affiliations:** 1 Faculty of Medicine, Almaarefa University, Riyadh, SAU; 2 Department of Anesthesiology and ICU, Tanta University Hospitals, Tanta, EGY; 3 Critical Care Medicine, King Saud Medical City, Riyadh, SAU; 4 Department of Internal Medicine, King Saud Medical City, Riyadh, SAU; 5 Internal Medicine, King Saud Medical City, Riyadh, SAU

**Keywords:** hematology, predictors, icu, mortality, sickle cell disease

## Abstract

Background

Sickle cell disease (SCD) is the most common genetic blood disorder in Saudi Arabia. A limited number of studies have been conducted on SCD patients regarding their intensive care unit (ICU) admissions. We aimed to identify the cause of ICU admission in SCD patients and to identify predictors of mortality.

Methodology

We identified 64 patients with SCD, aged 14 years and older, who were admitted to the ICU of King Saud Medical City, Riyadh, Kingdom of Saudi Arabia, from January 1, 2017, to December 31, 2020.

Results

Acute chest syndrome was the most frequent primary diagnosis for ICU admission in 29 (45.3%) patients followed by vaso-occlusive crisis in 23 (35.9%) patients. Pregnancy in eight (12.5%) patients was the most prevalent co-existing condition. The median age was 29 years, with males comprising 45.3% and females comprising 54.7% of the study population. Arterial blood gas pH less than 7.2 on ICU admission (p= <0.001), hemodialysis support (p= 0.049), the use of vasopressors (p= 0.016), intubation (p= <0.001), and being intubated within the first 24 hours of ICU stay (p= 0.04) had a statistically significant association with mortality at ICU discharge out of all the variables tested. Mortality on ICU discharge was 7 (10.9%).

Conclusion

This was a retrospective study carried out in King Saud Medical City. Comparing the results of the study to those of similar ones conducted around the world revealed a low SCD ICU mortality rate. This low mortality may be a result of improved overall ICU care. We recommend a multi-center, prospective study in future.

## Introduction

Sickle cell disease (SCD) is a genetic disorder responsible for the presence of an abnormal type of hemoglobin, hemoglobin S (Hb S). SCD occurs chiefly in individuals of African or Mediterranean origin [1]. Occasionally, Hb S polymerizes within the red blood cells (RBCs), blocking the small blood vessels and leading to vaso-occlusive events (VOEs), which are marked by ischemia and excruciating pain. Recurrent infarctions eventually lead to chronic organ failure. This process is referred to as acute chest syndrome (ACS) in the lung. In the world, SCD affects roughly 300,000 newborns per year [1]. Given the frequency of sickle cell anemia, it is expected that by 2050, there will be roughly 404,200 newborns with the disease [2]. SCD causes acute and chronic illnesses and lowers median life expectancy by at least 30 years worldwide. There is a vast range of severity, with some people exhibiting no symptoms and others encountering several, permanently altering effects [3]. ACS, infection, stroke, and end-stage organ failure are the leading causes of mortality [4,5].

SCD is among the most common genetic blood disorders in Saudi Arabia. The prevalence of SCD (4.5%) and β-thalassemia (1.8%) are both quite high in Saudi Arabia. The majority of these positive results (93.9% for SCD and 97.3% for β-thalassemia) were carriers [6]. The Eastern province of Saudi Arabia has the highest prevalence of SCD, while the southern regions have the second-highest prevalence, with the disease being widespread throughout the country [7]. In a study conducted at the Salmaniya Medical Complex in Bahrain, 210 SCD patients were admitted to the intensive care unit (ICU) during the study period. ACS was the main reason for the admission to the ICU. In the ICU, patients with SCD had a mortality rate of 12.7% [8]. In a study conducted at King’s College Hospital, London, UK, ACS (14, 30%) was the most common reason for admission among the 46 admissions to the critical care unit (CCU) throughout the study period. During the study period, SCD patients' CCU mortality was 19.6%, which was similar to the CCUs' overall mortality rate of 17.6% [9]. In another study conducted in France, 488 SCD patients were admitted to ICU. The main reason for ICU admission was ACS (47.5%). Sixteen (3.3%) patients died in the ICU, mainly of multi-organ failure following a painful VOE or sepsis [10]. In a study conducted at Detroit Medical Center, 108 patients with SCD were admitted to the medical intensive care unit; the commonest primary diagnosis for admission was ACS in 27 (25%) patients, and the mortality on discharge was 12.9% [11]. In a study done in Oman, 49 SCD patients were admitted 56 times to the ICU. The commonest reason for admission was ACS (69.6%). The mortality for SCD patients in their ICU was 16.1% [12].

SCD has a significant social and cultural impact on our country since it primarily affects young people, and their deaths are often sudden [13-16].

This study aims to provide insight into a major problem in the community due to a high prevalence of SCD in Saudi Arabia. Our study would help contribute to more preventive measures and better care of SCD patients who are admitted to the ICU. There are limited numbers of studies conducted on SCD patients regarding their ICU admissions worldwide. Thus, this study was conducted to identify the cause of ICU admission in SCD patients and to identify predictors of mortality in order to aid doctors presiding on such cases to recognize patients at elevated risk of mortality.

## Materials and methods

Subjects and methods

This was a retrospective descriptive study that included all adult patients with confirmed homozygous SCD, SCD, and sickle β0/β+ thalassemia admitted to the ICU of King Saud Medical City (KSMC), Riyadh, Kingdom of Saudi Arabia, between January 1, 2017, and December 31, 2020, excluding all patients younger than 14 years old and with sick cell trait. In case of multiple admissions, only the first admission was used.

Data of the patients were extracted from the KSMC ICU database, electronic medical record (EMR), and patient paper notes regarding baseline characteristics, date of admission, reason for admission to ICU, selected clinical and laboratory parameters, comorbidity, medication history and, therapeutic intervention during ICU stay. The primary outcome of interest was collected from the EMR: survival status upon discharge from ICU. Baseline laboratory values were collected from the most recent outpatient visit before ICU admission.

Statistical analysis

Microsoft Excel was used for data entry and management. SPSS Version 26 (IBM Corp., Armonk, NY) was used for data analysis, with a p-value of less than 0.05 considered significant. Fisher’s exact test was used for the exploratory analysis to look for association between APACHE (Acute Physiology and Chronic Health Evaluation) II score with survival status upon discharge from the ICU. Means and standard deviation were recorded for continuous variables for descriptive analysis.

Ethical consideration

Ethical approval from the Institutional review board (IRB) of KSMC (IRB registration number: H-01-R-053) was met before data collection began, and the purpose of the study was clearly explained to the institution. Regarding confidentiality and privacy, the personal information (name and contact information) of patients was not included since we used the medical records; only informed consent was waived.

## Results

The study included 64 patients with SCD who were admitted to the ICU of KSMC from January 1, 2017, to December 31, 2020. ACS was the most frequent primary diagnosis for ICU admission in 29 (45.3%) patients followed by vaso-occlusive crisis in 23 (35.9%) patients. Pregnancy in eight (12.5%) patients was the most prevalent co-existing condition. The median age was 29 years, with males comprising 45.3% and females comprising 54.7% of the study population. Mortality on ICU discharge was 7 (10.9%) (Table 1).

**Table 1:** Clinical characteristics analysis *Others include severe anemia, severe menorrhea, hemolytic crisis, stroke, post-CS, arrhythmias, and so on. ER, emergency room; ICU, intensive care unit; CS, cesarean section

Variable		Number (%)	Median (range)
Gender	Male	29 (45.3%)	
	Female	35 (54.7%)	
Age			29 (14-56)
Diagnosis on admission to the ICU	Acute chest syndrome	29 (45.3%)	
	Vaso-occlusive crisis	23 (35.9%)	
	Sepsis	5 (7.8%)	
	Others*	7 (10.9%)	
Route of admission to the ICU	ER	48 (75%)	
	Ward	14 (21.9%)	
	Referral	2 (3.1%)	
Mortality		7 (10.9%)	

Table 2 shows that the median hemoglobin level of the patients on admission to the ICU was 8.95, ranging from 3.6 to 12.9, and most of the patients had a pH of more than 7.2 on admission to the ICU.

**Table 2:** Descriptive analysis of admission variables ICU, intensive care unit

Variable		Number (%)	Median (range)
Hemoglobin levels on admission to the ICU			8.95 (3.6-12.9)
pH on admission	≥7.2	57 (89.1%)	
	<7.2	7 (10.9%)	

Table 3 shows that the median duration of ICU stay was 3.5 days, with a range of 1 to 38 days. Overall, 15 (23.4%) patients were intubated during their ICU stay, and 11 of them were intubated during the first 24 hours of their ICU admission. The median intubation duration was 2.5 days, with a range of 1 to 22 days. Five (7.8%) patients who were not on regular renal replacement therapy started hemodialysis at some point during the ICU stay, eight (12.5%) patients received vasopressors during their ICU stay, eight (12.5%) patients received non-invasive ventilation, and 12 (18.8%) patients had red blood cell exchange.

**Table 3:** Descriptive analysis of ICU variables ICU, intensive care unit

Variable	Number (%)	Median (with range)
Duration of stay in ICU (days)		3.5 (1-38)
Duration of intubation in the ICU (days)		2.5 (1-22)
Mechanical ventilation	15 (23.4%)	
Intubation within the first 24 hours of ICU admission	11 (17.2%)	
Renal replacement therapy	5 (7.8%)	
Vasopressors	8 (12.5%)	
Non-invasive ventilation	8 (12.5%)	
Red blood cell exchange	12 (18.8%)	

**Figure 1:**
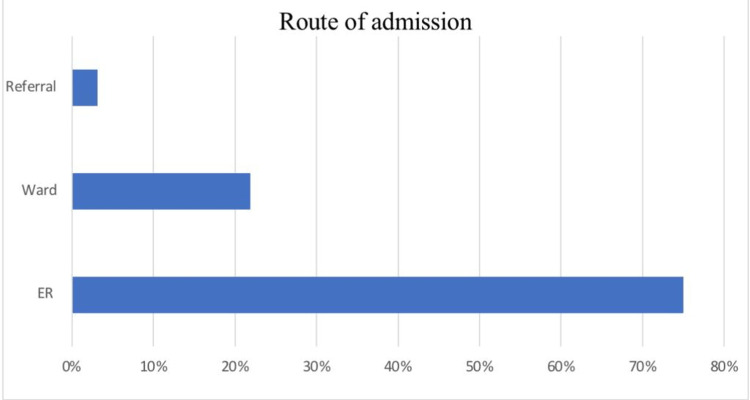
Percentage of the route of admission of study subjects ER, emergency room

Figure 1 shows that most (48, 75%) of the patients were admitted to the ICU through the emergency room (ER), 14 (21.9%) through the ward, and two were referred from other hospitals.

Table 4 shows that only arterial blood gas pH of less than 7.2 on ICU admission, hemodialysis support, use of vasopressors, intubation, and being intubated within the first 24 hours of ICU stay had a statistically significant association with mortality at ICU discharge out of all the variables tested.

**Table 4:** Analysis of variables associated with ICU outcome ER, emergency room; ICU, intensive care unit

Variable	Levels	Alive	Died	P-value
Gender	Male	25	4	0.505
	Female	32	3	
Route of admission	ER	44	4	0.339
	Ward	11	3	
	Referral	2	0	
Intubation	No	49	0	<0.001
	Yes	8	7	
Intubation within the first 24 hours of ICU admission	No	53	0	0.04
	Yes	4	7	
pH on admission	≥7.2	54	3	<0.001
	<7.2	3	4	
Vasopressors	No	54	2	0.016
	Yes	3	5	
Hemodialysis/continuous renal replacement therapy	No	53	3	0.049
	Yes	2	3	
	Chronic hemodialysis	2	1	
Non-invasive ventilation	No	49	7	0.289
	Yes	8	0	
Red blood cell exchange	No	46	6	0.748
	Yes	11	1	

Analysis was carried out for some admissions that had enough data to calculate APACHE II (41 admissions). The mean APACHE II score was 21.35, with a standard deviation of 6.3. A statistically significant correlation existed between an APACHE II score of greater than 25 and ICU death (p=0.002).

## Discussion

This study reviewed 64 patients with SCD who required admission to the ICU of KSMC. This group comprised of 35 females and 29 males, with their median age being 29 years. ICU mortality was 10.9% mainly of multi-organ failure, lower than in comparison to other studies conducted in Bahrain and the UK, which respectively showed an ICU mortality rate of 12.7% and 19.6% [8,9]. In a study conducted in France, the overall ICU mortality of SCD patients was 3.3% (16 out of 488). However, only 81 (16.6%) patients required life-supporting treatments (NIV, MV, vasoactive drugs, and renal replacement therapy). Out of these 81 patients, 16 (12.96%) died [10].

Out of the 64 SCD admissions, ACS was the most frequent primary diagnosis in almost half of the ICU admissions (29, 45.3%), followed by vaso-occlusive crisis in 23 (35.9%) patients and then sepsis in five (7.8%) patients. This was also in concurrence with a similar nationwide study conducted in France, which also showed that ACS accounted for most of the ICU admission at 47.5%, followed by VOE at the rate of 21.3% and then sepsis at 8.6% [10]. This also was in correlation with other studies conducted in the USA and Oman [11,12].

SCD admission to the ICU comprised of 15 (23.4%) patients who required intubation throughout the duration of ICU care, with a median intubation duration of 2.5 days with a range of 1-22 days, slightly less than that of the study conducted in the USA where 32 (29%) patients required mechanical ventilation with a median duration of 3 days with a range of 1-40 days [11].

Of the 11 patients in our study who required intubation within the first 24 hours of ICU admission, seven patients were pronounced deceased mainly due to multi-organ failure. A study conducted in the USA similarly showed that 11 patients required intubation within the first 24 hours, out of which nine died. Intubation within the first 24 hours was a mortality risk factor. This also concurred with the study conducted in Oman, where it showed an 89% mortality rate with requirement of mechanical ventilation [12]. Aligning with this, our results also showed a good predictor of mortality was having a pH level lower than 7.2. Of the seven patients whose pH was less than 7.2, only three survived. Similar results were found in a study conducted in the USA, which showed an association between pH less than 7.2 and mortality on ICU discharge [11].

In contrary to our study, the study conducted in Oman found that 46 (83%) patients admitted to the ICU were from the ward [12], and another study conducted in France found that 238 (48%) patients were admitted through the ward [10]. As per our study, most admissions predominantly came through emergency department (ED), 48 (78%), and ward admissions were considerably lower than studies mentioned above at a rate of 14 (21.9%) patients. This could reflect improved overall ward care, better treatment of sepsis, and multi-disciplinary approach to the management of these patients. This high SCD admissions to ED could be attributed to patients' inability to regularly follow up or poor compliance on patients' behalf, leading to high SCD admissions to ED. If that turned out to be the case, then taking positive proactive measures could have a significant effect on decreasing SCD admissions to the ICU, indirectly decreasing overall mortality.

Some already known risk factors were not found in the ICU database for all the patients and thus were excluded from the study, for instance, history of immunization, history of splenectomy, low baseline Hb F, elevated NT-proBNP (N-terminal pro-brain natriuretic peptide), elevated sVCAM-1 (soluble circulating vascular cell adhesion molecule-1), and severity of hemolytic anemia [17,18]. These studies were not conducted in the ICU settings.

There are some limitations that should be considered. Firstly, this study was a retrospective analysis, and, as such, the data collection was reliant on medical records. This could potentially lead to inaccuracies in the data, as not all the data points may have been accurately recorded. Secondly, this study was conducted in a single center, which may limit the generalizability of the results. The prevalence of SCD, the patient population, and the management practices may vary across different regions, making it difficult to apply these findings to other settings. Thirdly, the sample size of this study was relatively small, with only 64 patients included. This may limit the statistical power of the analysis and could lead to potential biases in the results.

## Conclusions

This was a retrospective study carried out in KSMC and focuses on identifying predicators of mortality among SCD patients admitted to the ICU between 2017 and 2020. This study revealed that the majority of SCD ICU admissions are due to ACS. The variables investigated that exhibited a statistically significant correlation with mortality at ICU included arterial blood gas pH of less than 7.2 on ICU admission, hemodialysis support, the use of vasopressors, intubation, and being intubated within the first 24 hours of ICU stay. Comparing the results of the study to those of similar ones conducted around the world, it also revealed a low SCD ICU mortality. This low mortality may be a result of improved overall ICU care. We recommend a multi-center prospective study in future.
